# Modeling timelines for translational science in cancer; the impact of technological maturation

**DOI:** 10.1371/journal.pone.0174538

**Published:** 2017-03-27

**Authors:** Laura M. McNamee, Fred D. Ledley

**Affiliations:** 1 Center for Integration of Science and Industry, Bentley University, Waltham, Massachusetts United States of America; 2 Department of Natural & Applied Sciences, Bentley University, Waltham, Massachusetts United States of America; 3 Department of Management, Bentley University, Waltham, Massachusetts United States of America; Iowa State University, UNITED STATES

## Abstract

This work examines translational science in cancer based on theories of innovation that posit a relationship between the maturation of technologies and their capacity to generate successful products. We examined the growth of technologies associated with 138 anticancer drugs using an analytical model that identifies the point of *initiation* of exponential growth and the point at which growth slows as the technology becomes *established*. Approval of targeted and biological products corresponded with technological maturation, with first approval averaging 14 years after the *established* point and 44 years after *initiation* of associated technologies. The lag in cancer drug approvals after the increases in cancer funding and dramatic scientific advances of the 1970s thus reflects predictable timelines of technology maturation. Analytical models of technological maturation may be used for technological forecasting to guide more efficient translation of scientific discoveries into cures.

## Introduction

There has been extensive debate about the efficacy of investments made in cancer research since the 1970s and how to achieve better results in the future. There is little debate that these investments have produced unprecedented insights into cancer biology. This progress is evident in the near-exponential growth in academic publications, as well as salient discoveries in oncogenesis, apoptosis, cancer immunology, genomics[1], and the emergence of targeted therapeutics[2], biologicals, cell therapies, and nanoparticles[3]. These investments have not, however, had temporal impacts on increasing annual anti-cancer drug approvals or survival for most major cancers[4, 5].

There is little theoretical understanding of why the benefits of cancer research have not been proportional to the rate of scientific progress. There are many opinions. A common view is that previous research has positioned science on the threshold of dramatic new breakthroughs in clinical care[5–7]. Some have proposed that the lack of progress reflects shortcomings in the structure of the scientific enterprise[8]. This view has spawned initiatives focused on: aligning research funding with disease burden[9]; collective formation, collaboration, and data sharing among researchers[10, 11], closer connections between “bench and bedside,”[5] greater emphasis on quality preclinical research[12], accelerated clinical trials of promising technologies[13], and reconsideration of the biopharmaceutical industry’s role in translational science[5, 14]. Others have argued that the emphasis on curing cancer is misguided, and that more research should be redirected at prevention[4, 15]. Some have even argued that the complexity of cancer represents an exceptional barrier for conventional therapeutics[16], or even that cancer represents an emergent property of a complex system that may not be amenable to reductionist understanding[17]. Each of these perspectives has addressed discrete elements of translation without providing a cohesive, theoretical framework for evidence-based improvement of this process. This work examines the connection between advances in cancer biology and development of new anticancer drugs through the lens of innovation theories that posit a relationship between the evolution of basic science and technology, and the capacity of these technologies to generate successful products.

Contemporary practices of research and development are often described in terms of a linear model of innovation which, in its simplest form, posits that the insights and inventions arising from pure basic science research provide the conceptual and material foundation for applied research and development, which leads to the emergence and commercialization of new products[18–20]. There has been extensive scholarly debate about the form, strengths, and weaknesses of this model both as a description of the translational process, as well as its utility in informing, or constraining, the progress of innovation[18, 21, 22].

Nevertheless, Godin has noted that “The model has been very influential. Academic organizations as a lobby for research funds and economists as expert advisors to policy makers have widely disseminated the model, or the understanding based thereon, and have justified government support of science using such a model.”[19] The concept of a progression from basic science to applications is reflected in the emphasis on the importance of basic research in the science establishment envisioned by Vannevar Bush in The Endless Frontier[19, 22, 23]. Bush wrote: “Basic research leads to new knowledge. It provides scientific capital. It creates the fund from which the practical applications of knowledge must be drawn.”[23] The Bayh-Dole Act provided a mechanism for this progression from basic research to practical applications of knowledge by establishing a legal process by which advances arising from government-funded, basic research in academic or government laboratories would be “transferred” to industry for subsequent development[24]. This progression is explicitly represented in the nine stages of the Technology Readiness Assessment, which is commonly required for Department of Defense (DOD), NASA, and other government contracts[25–27]. An analogous progression is also reflected in the conception of translational science as a series of phases beginning with T0, basic science discovery, and proceeding to T1, applied bench to bedside research, and then T2, the development of new products and clinical practice[28, 29]. This progression is also evident in the strategy, formulated in the 1970s, to focus investment aimed at curing cancer on “massive investment in basic science” based on “the assumption that unbiased fundamental research would hold the key to unlocking the secrets of cancer.”[1]

The work of Christensen and others have addressed the relationship between innovation and the advance of science and technology[30, 31]. Their work addressed the observation that there was often a profound lag in the emergence of successful products following radical scientific or technological insights or inventions[31–33]. The essential observation is that while nascent scientific insights and inventions arising from basic research embody considerable promise, products based on immature technologies commonly fail to achieve the performance standards required by existing markets. These studies also suggested that as technologies mature, they can reach a threshold required to meet, or surpass, the standards required by established markets[34, 35].

Various methods have been described for assessing and forecasting the growth of technologies[36]. The “technology readiness assessments,”[25–27] commonly used in the aerospace and defense industries, involve use of a rigorous scale for assessing the maturation of technologies, from the initial observation and reporting of basic principles to the operational proof of technological utility. Other methods involve qualitative, or judgmental, methods based on expert opinions, such as the often-used Delphi method, or quantitative analysis involving extrapolation, trend analysis, modeling, or simulation[36, 37]. An important insight into technology forecasting has been the observation that technology growth in many different sectors, such as semiconductors, communications, and transportation, exhibits a characteristic growth pattern, classically described as an S-curve[31–35, 38]. The S-curve can be divided into several phases; a nascent phase in which a new technology is initiated from precursor technologies by an insight or invention, a growing phase of exponential advance, and an established phase in which limits are encountered and advances slow. An extensive body of literature has examined the implications of the S-curve of technology growth for both sustaining and disruptive innovation and successful product development[31–33, 35, 38, 39].

As in other technology sectors, in biopharmaceutical development there is often a substantial lag between important scientific or technological advances and the emergence of new products. Cockburn and Henderson identified a 19-year lag between publications describing publicly funded “enabling discoveries” underlying 21 “high impact” drugs and first FDA approvals[40]. Toole demonstrated a 17–24 year lag between government funding for basic research and FDA approval of new drugs associated with that research[41]. A 2006 analysis of the impact of research funding by the Congressional Budget Office identified an 18-year lag between public funding of basic research and approval of new drugs based on this research, but did not examine reasons for this lag[42]. The work of Lichtenberg[43] addressed the lag between publications and improved cancer outcomes, concluding that published articles that cite research funding are more strongly correlated with clinical outcomes after 10 years than they are in contemporaneous years. A 2011 review by RAND Europe estimates that there was a 17-year lag between advances in basic science and product approvals based on this science, but also concluded “little is known about time lags and how they should be managed. This lack of knowledge puts those responsible for enabling translational research at a disadvantage.”[44]

Biomedical science provides few metrics for measuring the advance of technologies analogous to measures of chip density, computational or communication speed, or tensile strength used in other technology sectors[45]. Biopharmaceutical development, more than other technology sectors, is closely linked to basic science[46], and tends to be highly empirical in nature and less amenable to normative analysis[47].

We have previously investigated patterns of innovation in biotechnology using a bibliometric-based approach, which uses the accumulation of knowledge as a surrogate measure of scientific and technological advance[45]. Bibliometric methods have been used for S-curve modeling previously in sectors such as fuel efficiency, food safety, and optical storage[48] and have also been used to study the relationship between the growth of knowledge about specific cancers and reductions in the mortality rate[43]. Our initial study looked for qualitative patterns in the number of publications related to three novel biotechnologies—monoclonal antibodies, gene therapies, and nucleotide therapies—as well as subsets related to contributing technologies within each of these sectors. This work suggested that the cumulative number of publications had growth patterns consistent with those exhibited by performance metrics in other technology sectors with distinct nascent, exponential, and established stages. Most importantly, none of these three technologies generated successful products in the nascent or growing phases of technology growth of the S-curve. The first monoclonal antibody products came to market only after the technology reached an established phase, more than 20 years after the *initiation* and discovery of this technology[45], as did the first successful nucleotide therapeutic and approved gene therapy in the years after that report. Monoclonal antibodies matured through a series of technologies: from mouse proteins produced using hybridomas, to chimeric and humanized proteins, and eventually fully human proteins produced using recombinant technologies[45]. Similarly, nucleotide therapeutics matured through the introduction of novel chemistries and gene therapies matured through successive viral vectors with sequence modifications to maximize both safety and gene expression[45, 49]. These works suggested that the ability of advancing biomedical technologies to generate products that met the market expectations for efficacy, safety, and quality, as defined by the FDA, exhibited patterns similar to those of other technology sectors.

**Fig 1:**
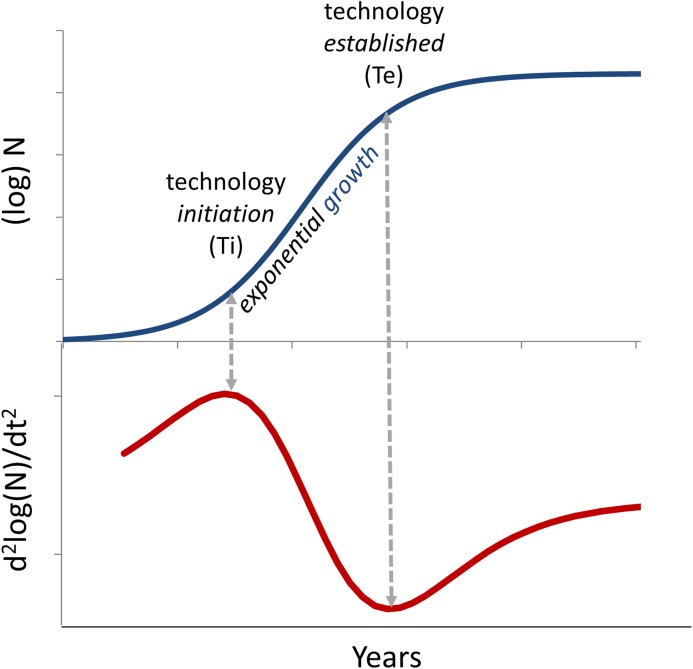
Quantitative model of the technology growth cycle S-curve. Growth of research in a specific area is modeled by a best-fit logistic equation (black line) fit to the log of the number of publications identified by a PubMed search. New areas of research emerge into a *nascent* stage and mature through a near-exponential *growing* stage before becoming *established* as limits are encountered. The rate of acceleration of the best fit model (red line) (i.e., d^2^y/dx^2^) is used to identify the *initiation* (Ti), representing the point of maximum exponential acceleration, and when the technology is *established* (Te), representing the point of maximum exponential deceleration of cumulative publications.

We have developed a numerical model for the growth of biomedical technologies called the Technology Innovation Maturation Evaluation (TIME^tm^) model, which examines the relationship between the growth of technology in selected research areas, and successful development of biopharmaceutical products based on those technologies[49]. In other technology sectors, the S-curve of technology growth is commonly modeled using various forms of the generalized logistic function[50–53]. The TIME model uses the cumulative number of entries in PubMed.gov for a specific technology as a metric for the accumulation of knowledge and growth of that technology. We have investigated the fit between the accumulated number of entries related to a specific technology and a number of logistic functions. We concluded that an exponentiated logistic function provided the most consistent fit to the cumulative number of publications and concordance between the numerically defined *initiation* point and seminal advances in the field noted in historical reviews. The TIME model is based on the use of the best-fit, exponentiated logistic function (see Materials and Methods), from which it is possible to calculate the point of maximum acceleration of publication accumulation, defined as the point of *initiation* for that technology, and the point of maximum deceleration of publication accumulation, defined as the *established* point for that technology (Fig 1).

The TIME model has also been used to examine patterns of innovation underlying all of the New Molecular Entities (NMEs) approved by the FDA 2010–2014. This study showed that 63/87 (72%) of NMEs discovered by targeted strategies were approved only after the *established* point of research on the drug’s target and that the median time from *initiation* to first approval was 36 years. This study also showed that the time in clinical development was significantly longer for NMEs that entered clinical development before technologies were *established* than for NMEs that entered clinical development after this point (11.3 years versus 8.4 years, p<0.001). In contrast, there was no association between the approval of NMEs discovered using phenotypic screening methods and metrics describing the growth of research on the drug target. This result is consistent with the expectation that discovery and development of phenotypic products are not dependent on advanced knowledge of a specific target or pathway[2, 54].

The present study examines patterns of innovation for the advent of new anticancer drugs, focusing explicitly on the lag in the emergence of new drugs in response to increases in cancer research funding in the 1970s. Our hypothesis is that the emergence of new anticancer drugs follows a characteristic pattern of innovation in which successful products emerge only after research on the enabling technologies achieves a certain level of maturation, and that the lag in new drug approvals corresponds to the time required for the scientific advances of the 1970s to reach this established point.

To test this hypothesis, we used the TIME model to examine the growth of technologies related to major classes of cancer drugs, and examined the relationship between the growth of these technologies and approval of drugs for treating cancer in each of these classes. This analysis quantifies the time lag between *initiation* of new areas of research to approval of drugs associated with this research, as well as the temporal relationship between maturation of this research and drug approvals.

## Materials and methods

### Data sources

Approved anticancer drugs and dates of first approval were identified from the National Cancer Institute (NCI) (http://www.cancer.gov/cancertopics/druginfo/alphalist), Tufts Center for Study of Drug Development[55], and drugs@FDA (http://www.accessdata.fda.gov/scripts/cder/drugsatfda/index.cfm). This analysis excluded cancer vaccines, anti-emetics, bone marrow stimulating agents, and cardioprotective agents. Drugs were classified as “biologic,” “phenotypic,” or “targeted” as described[2, 56]. The complete list of drugs is provided in S1 Fig.

NCI appropriations were identified at www.nih.gov/about/almanac/appropriations/index.htm. Appropriations were corrected for the Consumer Price Index at www.usinflationcalculator.com/inflation/consumer-price-index-and-annual-percent-changes-from-1913-to-2008/ and do not include $1.26B in supplemental funding from the American Recovery and Reinvestment Act.

### Analytical model

Bibliometric methods are commonly used to characterize the emergence of new technologies[45, 49, 57–61]. The use of bibliometrics in technology forecasting has been particularly common in the pharmaceutical industry because of its proximity to basic scientific results[62]. The TIME model quantifies the accumulation of knowledge by considering each publication about a specific technology as a quantum of new knowledge. The information value of any single publication may vary in magnitude ranging from large advances that contribute positively to knowledge, to incremental, inconsequential, or even incorrect (negative) contributions. However, when integrated over very large numbers of publications, the cumulative number of publications provides a quantifiable metric for the accumulation of knowledge[45, 63].

Publications were identified in PubMed.gov using search terms shown in S1 Table. Searches used MeSH terms when possible, and were restricted to publications associated with cancer using the Boolean function: AND “neoplasms”[MeSH Terms].

Many potential sources of measurement error exist in bibliometric analysis including, but not limited to, the efficiency of ascertaining relevant publications through a text-based search, changes in the lexicon of fields over time, the ascertainment of irrelevant publications containing coincident text strings, and errors arising from the process of adding metatags and archiving publications in the PubMed.gov database. The use of MeSH terms for search was designed to take advantage of the quality controls built into MeSH assignments and the non-ascertainment of publications due to changes in lexicon[64].

The S-curve of the technology growth cycle is modeled using the logistic equation:

Y*=L/(1+e^(−(mx+b))

where Y* is the log of the cumulative number of papers, x is years, and L the limit of Y*. L represents the log of the predicted maximum number of papers if progress continues along a typical S-curve. Parameters were determined using a Fisher-Pry transformation of the bibliometric data[65]. The second derivative of the best-fit logistic equation is used to identify the *initiation* point, representing the point of maximum exponential acceleration of cumulative publications (max d^2^y/dx^2^), and the *established* point, representing the point of maximum exponential deceleration of cumulative publications (min d^2^y/dx^2^). Errors for parameters (Ti, Te) were estimated based on the residuals of the best fit curve.

Validation studies have shown that the *initiation* point corresponds with seminal scientific or technological advances that initiate new areas of research and exponential growth in publication activity (see Results). The *initiation* point thus corresponds to the beginning of the T0 or discovery research phase of translational science[29, 66, 67]. The interval between the *initiation* point and NME approval represents the time between a scientific discovery or invention, and launch of a drug based on these advances, corresponding to the T0 + T1 + T2 phases of translational science[29, 66–68].

## Results

### Defining the lag in emergence of drugs for cancer therapy

**Fig 2:**
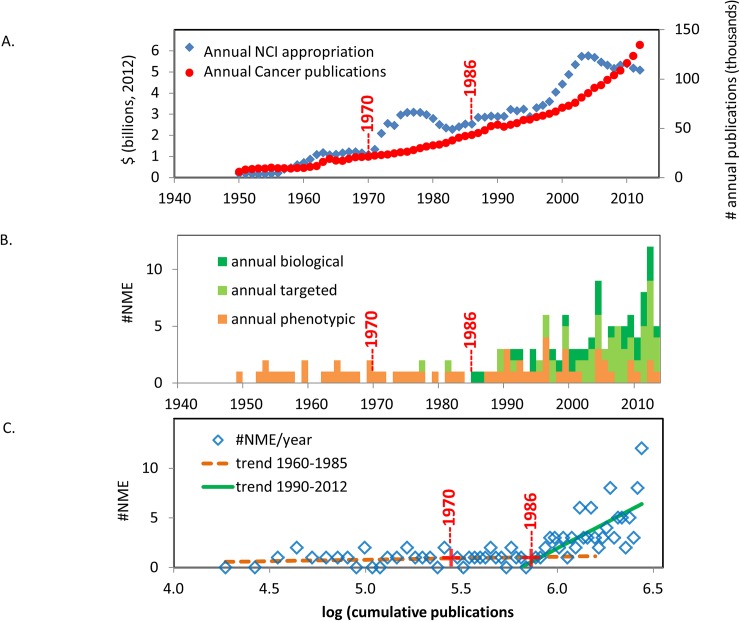
(A) Annual appropriations for the National Cancer Institute (NCI) and publications related to cancer, 1950–2012. NCI appropriations are shown in constant 2012 dollars and exclude supplemental funding from the American Recovery and Reinvestment Act in 2009–2010. Publications on cancer were identified in PubMed.gov using the search term “neoplasms”[MeSH]. (B) Annual approvals of anticancer drugs. Approved drugs are classified as biologic, phenotypic, or targeted based on the composition of matter or method of drug discovery as described[56]. (C) The relationship between publications on cancer (log scale) and annual approvals. Trend lines are shown for 1960–1985 (not significant) and 1985 to the present (p<0.005). Data points 1970 and 1986 are indicated for reference only.

There were >2.7 million publications identified by “neoplasms”[MeSH] between 1950 and 2012, an interval in which there was >$170 billion in NCI appropriation (constant dollars) (http://www.nih.gov/about/almanac/appropriations/index.htm.)) (Fig 2A). A dramatic increase in funding for the NCI is apparent in the early 1970s following the signing of the National Cancer Act of 1971. Additionally, there is a steep increase in NCI funding in conjunction with a general increase in government funding for biomedical research in the late 1990s. A 1999 report prepared for the Institute of Medicine estimated that NCI funding represented approximately half of total funding for cancer research provided by government, industry, and various foundations[69]. There is a high correlation between funding and annual publications from 1950 and the present (0.92, p<0.0001).

A total of 138 NMEs, including both small molecules and biologics, were approved from 1999–2013 (Fig 2B). Of the 138 NMEs, 65 were small molecules discovered through phenotypic screening, 47 were small molecules discovered through targeted methods, and 26 were biologics. There was little change in the number of NME approvals from 1950 to the late 1980s. This pattern changed with the emergence of increasing numbers of targeted and biologic therapeutics in the late 1980s and 1990s. Fig 2C shows the annual number of drug approvals as a function of cumulative publications on cancer. From 1950–1985, there was no significant correlation between the number of new drug approvals and the >700,000 publications identified by “neoplasms”[MeSH] (1950–1985 R^2^ = 0.06, NS). Since the late 1980s, there has been a significant correlation between the increasing NME approvals, dominated by targeted and biological products, and cumulative number of publications (1986–2012 R^2^ = 0.59, p<0.01). The 16-year interval between increases in funding for cancer research and increases in the number of NME approvals for cancer therapy is consistent with earlier observations of the lag between research funding and new drug approvals by the Congressional Budget Office, RAND, or others[42, 44].

### Growth cycles of technologies associated with discovery and development of cancer drugs

Sixteen technologies associated with the discovery or development of new anticancer drugs were selected based on: (i) the classification of anticancer drugs by Martell et al., including: antimetabolites (including antifolates), alkylating agents, alkaloids (including anti-microtubules), anthracyclines, estrogen/progesterone (hormonal), topoisomerases, cancer immunology, oncogenes, protein kinases, epigenetics, and apoptosis[70]; (ii) selected enabling technologies including recombinant proteins, monoclonal antibodies, genomics, systems biology, and synthetic biology; (iii) and the ability to discretely identify relevant publications in sufficient numbers for statistical analysis[70]. Each of these technologies represents broad areas of investigation in basic and applied science, drug development, and oncology, as opposed to investigations related to any specific target or candidate product.

Of the sixteen technologies, thirteen exhibited a characteristic growth pattern in which the log of the cumulative number of publications exhibited a logistic pattern of growth and could be modeled with the TIME model (S1 Fig). Research areas that cannot be modeled with the TIME model include nascent technologies currently experiencing an exponential growth phase that have not yet approached a limit, technologies that exhibit complex growth patterns indicative of a series of sequential S-curves, and some technologies that exhibit inexplicable patterns. The three technologies that could not be modeled in this study reflect contemporary areas of research (epigenetics, systems biology, and synthetic biology), which exhibited a pattern of exponential growth consistent with technologies that have not yet approached an *established* point. We would note that a current limitation of the TIME model is that the numerical methods that are used do not enable prediction of when exponential growth may slow, and whether these technologies will ultimately exhibit a characteristic S-curve pattern of growth.

For technologies that could be quantified with the TIME model, the *initiation* point was calculated as the point of maximum exponential acceleration, and the *established* point was calculated as the point of maximum exponential deceleration. To validate the relevance of the numerically defined *initiation* point, we compared this date with key milestones identified in review articles for each technology. For certain technologies, the *initiation* point corresponded to the date of specific technological advances within that technology field. For example, the i*nitiation* point for monoclonal antibodies corresponded with the work of Cotton, Kohler, Milstein, Jerne, and others circa 1973–1975 on myelomas and hybridomas that enabled production of monoclonal antibodies, and the *initiation* point for recombinant proteins corresponded with the work of Cohen, Boyer, Berg, Nathans, Smith, and others circa 1970–1973 that enabled production of recombinant proteins. For other technologies, the *initiation* point corresponds with scientific insights, rather than discrete inventions. For example, the *initiation* point for oncogenes and tyrosine kinases corresponded with the convergence of research on protein kinases and research on oncogenic retroviruses, which led to recognition of oncogenes and tyrosine kinases. Similarly, the *initiation* point for apoptosis corresponded with studies on programmed cell death in embryogenesis, which led to an understanding of the role of apoptosis in cancer in the 1980s. For other technologies, the *initiation* point corresponded to studies on natural products that uncovered novel pathways. For example, the *initiation* point for research on microtubules in cancer corresponded with early studies on the mechanism of action of vinca alkaloids, the *initiation* point for research on topoisomerase corresponded with early research on the mechanism of action of epipodophyllotoxins, and the *initiation* point for research on histone deacetylases and epigenetics corresponded with research on the mechanism of action of trichostatin.

### Timelines from *initiation* of research to new drug approvals

Of 16 selected technologies, 12 were associated with at least one approved drug, and 118/138 approved anticancer drugs were associated with at least one of the selected technologies. Drugs not directly associated with one of the selected technologies were prednisone, thalidomide-related compounds (3 drugs), antibiotics (2 drugs), asparaginase (2 drugs), platinums (3 drugs), proteasome inhibitor (1 drug), adjuvants (2 drugs), protein synthesis inhibitors (2 drugs), hedgehog pathway inhibitors (1 drug), and miscellaneous (3 drugs).

**Fig 3:**
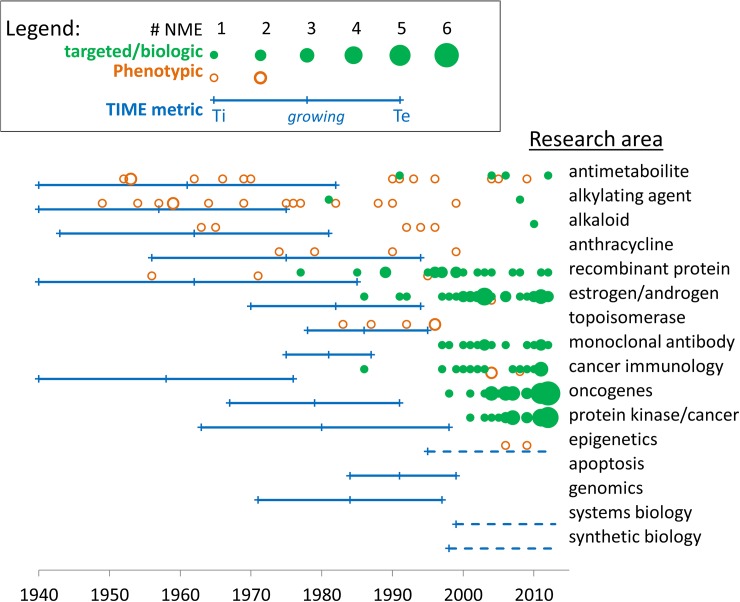
The relationship between drug approval dates and growth of associated technologies. Timelines for the growth of technologies are shown as blue bars from the *initiation* point (Ti), through the exponential *growing* stage, to the point at which the technology is established (Te). The number of approvals associated with each technology is shown for phenotypic drugs (open, orange circles) and targeted or biologic products (closed, green circles). Approval dates for drugs associated with multiple technologies are shown more than once.

**Fig 4:**
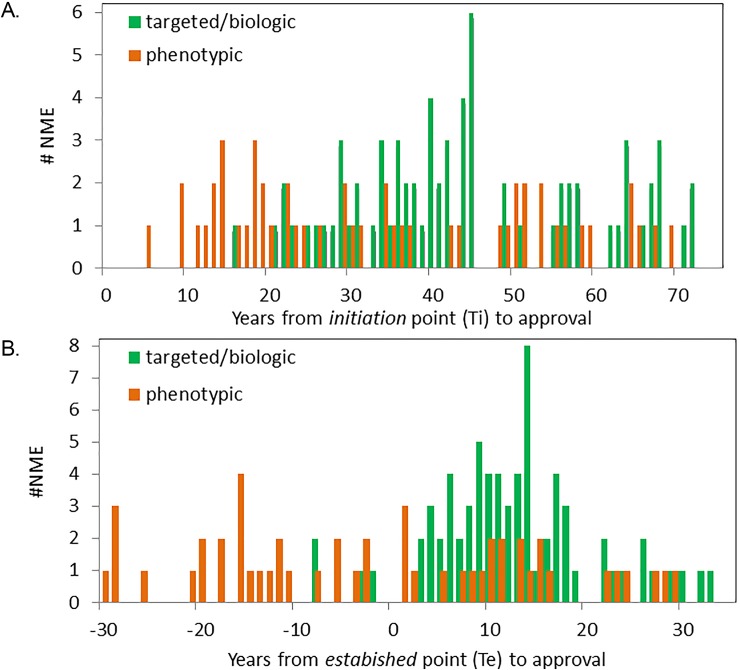
Timelines of drug approval and growth of associated technologies. (A) Years from *initiation* of associated technology (Ti) and approval of phenotypic drugs (orange bars) and biologic and targeted products (green bars). (B) Years from technologies becoming established (Te) and approval of phenotypic drugs (orange bars) and biologic and targeted products (green bars). Each drug is shown only once in association with the Ti and Te of the lagging technology.

Fig 3 shows the temporal relationship between the exponential *growing* phase of the curve, from *initiation* (Ti) to *established* (Te), and approval of associated drugs. Sixty-eight percent (44/65) of phenotypic drugs (open circles) were approved prior to the *established* point of technology growth. These phenotypic drugs include many antimetabolites, alkylating agents, and anthracyclines as well as phenotypically-discovered natural products that inhibit topoisomerase and histone deacetylase. In contrast, only 14 percent (10/73) of biologic or targeted products were approved before the associated technologies passed the *established* point, while 86% (63/73) were approved in the years after the *established* point. There was a significant difference between the timelines for development of phenotypic NMEs versus targeted or biologic NMEs as measured by the interval between *initiation* point and FDA approval (44 years versus 34 years, p<0.001) (Fig 4A), or the interval between the technology being *established* and approval (13 years versus -2 years, p<0.001) (Fig 4B). This result is consistent with the fact that discovery and development of targeted and biologic products are explicitly predicated on accumulated knowledge of the drug target or biologic product, while phenotypic development often proceeds without complete knowledge of the drug target or mechanism of action.

## Discussion

The expansion of funding for cancer research since the 1970s correlates not only with near-exponential growth in the number of publications, but also to a series of salient scientific discoveries that promised to provide new targeted and biologic therapies for treating cancer[5]. Nevertheless, for several decades after these increases in funding, there was no proportional increase in approval of NMEs. In this report, we examine the lag in emergence of NMEs for cancer therapy since the 1970s, building on theories of innovation that posit a relationship between technological maturation and the ability of technologies to generate successful products, as well as observations in many technology sectors that successful products emerge only after research on the enabling technologies achieves a certain level of maturation[31–33]. The data presented are consistent with the stated hypothesis that the emergence of new anticancer drugs follows a characteristic pattern of innovation in which successful products emerge only after research on the enabling technologies achieves a certain level of maturation, and that the lag in the emergence of NMEs represents a predictable latency associated with the time required for nascent science and technology to reach this level of maturation. There are several aspects to this observation.

First, using an analytical model for technology growth, we show that the emergence of targeted and biologic NMEs often follows normative patterns of innovation. For most of the technologies examined, technology growth, as measured by accumulation of publications, exhibited a characteristic S-curve growth pattern, with the technology *initiation* point introducing a stage of exponential growth that persisted until the technology reached an *established* point. Moreover, it was only after this *established* point that these technologies began to efficiently generate new targeted and biologic NMEs. Those technologies that did not exhibit an S-curve in this analysis exhibited an exponential growth pattern characteristic of nascent technologies, and none have generated approved NMEs. The characteristic patterns of technology growth and NME approvals observed in this study are similar to those we have observed in other studies including studies of drugs for Alzheimer’s disease and gene therapies[45, 49, 63], of which 80–90% of which exhibit the exponentiated logistic growth pattern that can be modeled with the TIME model.

While the accumulation of publications readily fits an exponentiated growth model, there is no reason to believe that the progress of research is best characterized by this monotonic picture. This analysis does not distinguish publications describing the discipline-specific contributions to technology growth arising from basic molecular, pathological pharmaceutical, preclinical, or clinical studies. Nor does this analysis distinguish the multiplicity of incremental or radical innovations that may be necessary to bring a complex pharmaceutical product to market, many of which occur independently or in parallel. Future studies should be directed at examining the contributions of individual components in the process of translational science, and how they contribute to the characteristic S-curve of publications associated with a discrete technology.

Second, for all of our accumulated studies, we have found that the large majority of targeted and biologic NMEs are approved only after the associated technologies pass the *established* point. These observations are consistent with the stated hypothesis that “the emergence of new anticancer drugs follows a characteristic pattern of innovation in which successful products emerge only after research on the enabling technologies achieves a certain level of maturity.” These observations are also consistent with the stated strategy of targeted and biological drug discovery, which is explicitly predicated on accumulated observations from biomedical research regarding the molecular mechanisms of disease, target identification and validation, and identification of drugs that impact these targets[56, 71]. In essence, science-based innovations are not occurring until the science base is mature.

In this context, the fact that no such association was observed between technology growth and approval of phenotypic NMEs is also significant. This observation is consistent with strategies for phenotypic drug discovery, which are explicitly predicated on observed biological effects, not an *a priori* understanding of disease mechanisms[56, 71]. We would also note that this analysis does not address the myriad innovations in medicine that may occur in the absence of scientific or technological advances through improvement in practice or those related to new medical devices, diagnostics, or accumulated know-how[72].

These observations do not establish causality between the accumulation of knowledge and successful development of targeted and biologic NMEs. Specifically, these observations do not rule out reverse causality. For example, it is possible that the slowing of publication growth, which defines the calculated *established* point in the TIME model, is a consequence of the progress being made towards approval of targeted and biological NMEs. Such a scenario would posit that the progress of lead products towards approval impacts, and reduces, the rate of accumulation of publications in the academic literature, or that there could be a feedback loop between these two processes. The fact that approval of phenotypic NMEs is not associated with the *established* point argues against progress towards approval, itself, being sufficient to cause of slowing in publication growth.

Other factors, not addressed in this study, could contribute to both the slowing of publication growth and progress towards approval of targeted and biological products. For example, Brown[73] has suggested that the methods used for targeted discovery may, themselves, be maturing along an S-curve analogous to that described by Christensen[31–33]. Cockburn and Henderson have suggested that pharmaceutical companies have an inherent “absorptive capacity” of the pharmaceutical companies, which can impact the relationship between scientific advances and NME approvals[40]. Toole has suggested that the level of R&D spending can also impact this relationship[41].

While widely used to assess the progress of scientific research, the accumulation of publications is a rather crude metric of scientific advance. As noted above, the cumulative number of publications subsumes research describing a number of critical milestones on the path to drug development. Eder et al. have highlighted publications related to target discovery and validation, demonstration of disease association, emergence of a therapeutic concept and mechanism of action, as well as lead and final molecule generation as milestones in the development of first in class products[74]. The publication record will also include research related to the metrics included in Lipinski’s “rule of 5,”[75] considered the gold standard for successful, small molecule drugs, as well as the knowledge base represented by Astra-Zeneca’s “5 R’s”[76] or analogous rubrics used in other organizations as predictors of drug development success. Further work will be required to differentiate the role of broad measures of technology growth, such as the TIME metrics and the role of individual milestones within this corpus.

Third, the present observations are also consistent with the hypothesis that the lag in new drug approvals corresponds to the time required for the scientific advances of the 1970s to reach this *established* point. If true, the lag in emergence of new drugs for cancer therapy is following a pattern also observed in many other technology sectors[30, 31, 38], which is associated with a number of strategies and tools for strategic management of technology including technology roadmapping, technological forecasting, and technology readiness assessments. These observations do not rule out other mechanisms that could contribute to this lag exerting their influence independently, or in consort, with the growth of technologies. For example, it has been suggested that methods for targeted drug discovery, which first emerged in the 1970s and 1980s, may, themselves, be maturing along an S-curve, and are only now reaching a level of maturation sufficient to compete with phenotypically discovered drugs[73]. Similarly, the structure of the scientific enterprise[8], the amount [41] and focus of research funding[9], connections among researchers[10, 11] and clinicians[5], the quality preclinical research[12], the involvement of industry[5, 14], the “absorptive capacity” of industry[39], and the nature of the available market[40] may all have either independent, or interactive, effects on this lag.

Further studies are needed to address the differential effects of such dynamics, and their interactions, on the lag in emergence of new drugs for cancer therapy. We have previously observed, for example, that capital investments in gene therapy were asynchronous with the growth of these technologies, as measured with the TIME model[49]. That study tracked >$5 billion in capital investments in gene therapy companies prior to 2012, and correlated the number and magnitude of investments with the growth of the gene therapy technology. We observed that the large majority of capital investments in gene therapy were made in technologies that had not reached the midpoint of the technology growth curve, and none of investments led to products[49]. We proposed that this “Asynchrony between the maturation of gene therapy technologies and capital investment in development-focused business models may have stalled the commercialization of gene therapy.”[49]

Similarly, a study of IPOs during the 2000 window showed an inverse relationship between capital investments and the maturation of each company’s core technology at IPO. We also observed that, despite this investment, none of the companies with nascent technologies successfully developed products based on these technologies, while companies with more mature technologies were able to develop a number of successful products[77]. These observations also pointed to the need for synchrony between technology maturation, investment, and clinical investigations in drug development[45, 49].

The observations in this report may have implications for translational science strategy. First, while the exponential acceleration of research output following a salient scientific discovery or invention often generates considerable excitement and, sometimes, unintended hype, this analysis suggests that such advances are less likely to generate products in the near term than to *initiate* the growth cycle of a new research area. In this context, the next wave of NMEs for cancer therapy may be more likely to come from technologies that are now reaching the *established* point, such as genomics, immunotherapy, or even gene therapy, than from technologies that are now in the nascent or growing phases of their growth cycles such as systems biology or synthetic biology.

Second, this analysis suggests that the timelines of translational science might be significantly shortened through initiatives that accelerate the growth of science and technology as well as the timelines of clinical investigation and regulatory review. Reichert and Wenger have reported that for anticancer drugs approved between 1990 and 2006, the average length of clinical trials was 74.4 months (6.2 years), and the combined clinical and regulatory phases averaged 84.2 months (7 years)[55]. For the same set of products, our data shows that the average time from *initiation* of the associated technology to drug approval was 41 years, and the average time between the technology reaching the *established* point and approval was 9 years. Thus, fractional reductions in the length of time required for maturation of technology could have a greater effect on accelerating drug approval than proportional reductions in the timelines for clinical trials or regulatory review. In fact, several initiatives are already directed at advancing the early stages of the translational process including a commitment to increase research funding in the recently-passed 21^st^ Century Cures Act[78], efforts to improve collaboration, communication, and data sharing[10, 11], the Accelerating Medicines Partnership[79], and efforts to advance preclinical science by the National Center for Advancing Translational Sciences[13].

Finally, the timelines of translational science for targeted and biological products estimated using the TIME model (including an average of 44 years from research *initiation* to approval and 14 years from research being *established* to approval) suggest the need for a long-term perspective on the societal impact of funding for basic cancer research. This analysis suggests that the impact of funding for cancer research should not be measured by near-term metrics of new drug approvals or clinical outcomes, nor should it be measured by the outcomes of clinical trials undertaken with *nascent* technologies. Rather, the impact of basic and applied research funding might be more appropriately measured by the advance of technologies through predictable growth cycles. Additionally, if these data do, in fact, reflect an essential characteristic of the relationship between the growth of research and timelines for the emergence of new drugs, these observations suggest that reductions in research funding and research activity might have deleterious impacts on timelines for new drug development and clinical outcomes that are not recognized for decades.

Finally, this analysis emphasizes the importance of examining patterns of innovation in translational science and developing analytical tools to guide strategic policy interventions aimed at accelerating this process. Recognizing recurrent patterns of innovation, and developing analytical tools for modelling these patterns, can have a positive impact on project management and policy formulation. Tools for technology road mapping[80],technological forecasting[36, 37], Technology Readiness Assessment, and Technology Maturation Planning[27] are routinely used for strategic management of technology by many industries. Such methods can play an important role in avoiding decisions based on the unrealistic expectations and “hype” surrounding many *nascent* scientific advances, battlefield views of impediments to progress, or the narrowly focused perspectives and objectives of multifarious stakeholders. The development and application of such tools in pharmaceutical development could be an aid to efficient allocation of R&D resources, prioritization of research projects, and the design of clinical trials with clinical endpoints and objectives that are appropriate for the stage of technology maturation. Such data-driven strategies might improve the efficiency of translational science, reduce the timelines and costs of product development, and more accurately inform the expectations for the future.

## Supporting information

S1 Fig TIME modeling of technology growth cycles for selected technologies. The log of the cumulative number of publications in PUBMED for each research area is shown as symbols. The best fit logistic regression is shown as a solid line in a corresponding color. Three technologies could not be modeled as a logistic regression and are shown as dotted lines connecting annual data. These technologies appear to be in the early, exponential phase of their growth cycle.
Publications were identified in the PUBMED database of the National Center for Biotechnology Information (NCBI) using the search terms shown in S1 Table. The S‐curve of the technology growth cycle is modeled using the logistic equation: Y* = L/(1+e^(‐(mx+b))) where Y* is the log of the cumulative number of papers (y*), x is years, and L the limit of Y*. L represents the log of the predicted maximum number of papers if progress continues along a typical S‐curve. The second derivative of the best-fit logistic equation is used to identify the initiation point (Ti), representing the point of maximum exponential acceleration of cumulative publications (maximum d2y/dx2), and the point at which the technology becomes established (Te), representing the point of maximum exponential deceleration of cumulative publications (minimum d2y/dx2).
(TIF)

S1 Table Search terms and TIME metrics for associated technologies. (XLS)
