# Supplementary figures and images for: Modeling timelines for translational science in cancer; the impact of technological maturation
Source: PLoS One. 2017 Mar 27;12(3):e0174538. doi: 10.1371/journal.pone.0174538 (PMC5367812; doi:10.1371/journal.pone.0174538)

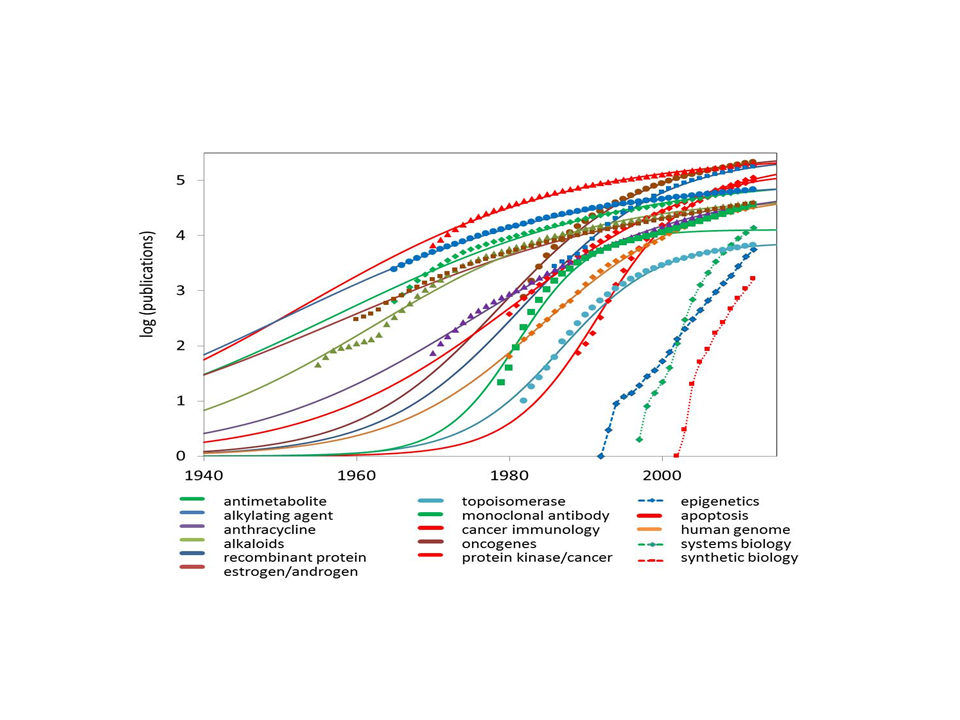

Supplement: S1 Fig — The log of the cumulative number of publications in PUBMED for each research area is shown as symbols. The best fit logistic regression is shown as a solid line in a corresponding color. Three technologies could not be modeled as a logistic regression and are shown as dotted lines connecting annual data. These technologies appear to be in the early, exponential phase of their growth cycle. Publications were identified in the PUBMED database of the National Center for Biotechnology Information (NCBI) using the search terms shown in S1 Table. The S‐curve of the technology growth cycle is modeled using the logistic equation: Y* = L/(1+e^(‐(mx+b))) where Y* is the log of the cumulative number of papers (y*), x is years, and L the limit of Y*. L represents the log of the predicted maximum number of papers if progress continues along a typical S‐curve. The second derivative of the best-fit logistic equation is used to identify the initiation point (Ti), representing the point of maximum exponential acceleration of cumulative publications (maximum d2y/dx2), and the point at which the technology becomes established (Te), representing the point of maximum exponential deceleration of cumulative publications (minimum d2y/dx2). (TIF) [file pone.0174538.s001.tif]
